# Design and content validation of a set of SMS to promote seeking of specialized mental health care within the Allillanchu Project

**DOI:** 10.1017/gheg.2017.18

**Published:** 2018-01-31

**Authors:** M. Toyama, F. Diez-Canseco, P. Busse, I. Del Mastro, J. J. Miranda

**Affiliations:** 1 CRONICAS Centre of Excellence in Chronic Diseases, Universidad Peruana Cayetano Heredia, Lima, Peru; 2 Instituto de Investigación Científica, Universidad de Lima, Lima, Peru; 3 Facultad de Salud Pública y Administración, Universidad Peruana Cayetano Heredia, Lima, Peru

**Keywords:** Developing countries, mhealth, SMS, text messaging, validation studies

## Abstract

**Background:**

The aim of this study was to design and develop a set of, short message service (SMS) to promote specialized mental health care seeking within the framework of the Allillanchu Project.

**Methods:**

The design phase consisted of 39 interviews with potential recipients of the SMS, about use of cellphones, and perceptions and motivations towards seeking mental health care. After the data collection, the research team developed a set of seven SMS for validation. The content validation phase consisted of 24 interviews. The participants answered questions regarding their understanding of the SMS contents and rated its appeal.

**Results:**

The seven SMS subjected to content validation were tailored to the recipient using their name. The reminder message included the working hours of the psychology service at the patient's health center. The motivational messages addressed perceived barriers and benefits when seeking mental health services. The average appeal score of the seven SMS was 9.0 (SD±0.4) of 10 points. Participants did not make significant suggestions to change the wording of the messages.

**Conclusions:**

Five SMS were chosen to be used. This approach is likely to be applicable to other similar low-resource settings, and the methodology used can be adapted to develop SMS for other chronic conditions.

## Introduction

Mental disorders are currently one of the most prevalent and disabling conditions worldwide, and have become an important issue for public health [1–2]. A similar scenario is found in Peru, where neuropsychiatric disorders are the leading cause of disease burden [3], and affect 1 in 5 Peruvians [4].

Despite its ever-increasing relevance, most healthcare systems have not adequately addressed this issue, and mental disorders continue to be underdiagnosed and undertreated [5, 6], creating what is currently known as a mental health treatment gap [7]. In low- and middle-income countries (LMIC), between 76% and 85% of patients with mental disorders do not access mental health care [7–9]. Similarly, results from epidemiological studies conducted by the Peruvian National Institute of Mental Health show that 69–85% of individuals do not seek mental health care, despite recognizing the need for it; with the main reasons stated for not seeking care being lack of financial resources and lack of information as to where seek care [10–14].

One of the main limitations to tackle the mental health treatment gap is the limited financial and human resources for mental health. Moreover, the few human resources available in LMIC have not been trained or are inefficiently distributed within the health system [7]. Technology, particularly mobile technology, has shown to aid the task-shifting process by providing tools for clinical decision-making and decision-support [15–18], data collection [17–19], as well as patient follow-up [17, 19]. More specifically, short message service (SMS) has been widely used in patient follow-up and disease management, showing improvements in medication adherence, symptom monitoring, attendance to appointments, and satisfaction with health services [17, 19–22], for conditions such as diabetes [23] and cardiovascular disease [24]. The use of technology specifically for mental health conditions appears promising, showing potential effectiveness of online, text messaging, and telephone support interventions [25].

Though the use of SMS in research has increased over the years, one of the limitations in the reporting of its use is the lack of information regarding the development process, and specifically the development of the content of the SMS [20, 26]. From a health communications point of view, such development is crucial because it not only reduces uncertainty about what behavioral changes could be expected but also identifies an efficient pathway of influence whereby messages appeal to cognitive mediators that are consistent antecedents of behavior change [27]. Thus, this process requires using theoretical frameworks, and in so doing, more chances of engagement and chance of the target behaviors will be expected [27]. The main objective of this paper is to describe the content design, development and content validation of a set of SMS used as part of the implementation of a mental health intervention at the primary care level in Peru.

## Methods

In this study, we report on the design and content validation of a set of SMS to support the linkage of participants that were screened for common mental health disorders with further mental health care at the primary care level. These activities were nested within the *Allillanchu* Project, an implementation project aimed to develop and test an intervention that combined the training of primary health care providers and the use of mobile health (mHealth) to improve the early detection, opportune referral and access to specialized treatment of people suffering from mental disorders and attending primary health care facilities in Lima, Peru. Within this intervention, the SMS component was a key element to promote access to specialized treatment.

### Description of the project's intervention

The mHealth element of the intervention consisted of an integrated system with three components: (a) an app containing a screening test for common mental disorders – depression, anxiety, psychosis, convulsive disorder, and alcoholism – to be used by primary health care providers during their regular consultations, (b) a Web-based data collection platform, used by the research team, designed to monitor the use of the app and the number of patients screened, (c) an SMS delivery app to dispatch the set of validated SMS to patients who screened positive for mental disorders.

During 9 weeks, individuals attending prenatal, tuberculosis, HIV/AIDS or chronic diseases services were screened for mental disorders by their health providers. The research team collected contact information of the patients. If the screening for any of the conditions of interest resulted positive, patients were advised to seek further specialized care and the Web-based data collection system automatically programmed and sent six SMS, three times per week, over a period of 2 weeks. The objectives of the messages were to remind the patient of the recommendation to seek mental health care and to motivate them to do so.

The text messages used in the intervention were developed in two phases: design and validation phase. The overall goal was to develop a valid final set of five SMS, i.e., four different motivational SMS and one reminder message to be sent twice.

### Phase 1: design of the SMS

#### Setting and participants

In order to develop the set of SMS within the framework of the *Allillanchu* Project, we aimed to collect information from patients attending public primary health care services similar to where the intervention of the project would be implemented. Therefore, we aimed to interview patients from prenatal care, tuberculosis, HIV/AIDS, and chronic diseases services.

In total, we initially aimed to interview 50 patients, 10 individuals per type of health condition (tuberculosis, HIV/AIDS, diabetes, and hypertension) and 10 pregnant women. Due to difficulties with finding an HIV/AIDS service at a primary health care center, this group of patients was not included in this phase, leaving a total of 40 patients to be interviewed.

Inclusion criteria included being 18 years or older, being able to provide informed consent, being a user of the primary health care service at the health center where the interview was to take place, being diagnosed at least 3 months prior to the interview or, in the case of pregnant women, being at least in the third month of pregnancy, and for patients with tuberculosis, having completed the second month of treatment and having a negative result in their sputum culture test.

#### Data collection tools and procedures

The SMS contents to be developed as part of the study were to be informative, appealing, and tailored to the potential recipients. Therefore, the development of the contents was to be based on perceived barriers, facilitators, and positive consequences of seeking psychological help as reported by patients from the health services where the project's intervention was implemented.

In order to achieve this, the research team, in collaboration with a communicator, with expertise in health communication, developed a 26-item questionnaire with open- and close-ended questions to collect information to design the SMS (see Supplementary Material 1). The 26 items were divided in four sections: (a) demographic data; (b) use of cellphones, to gain insight about mobile phone literacy and previous experience using SMS; (c) opinion of the SMS component of the *Allillanchu* project, to explore the willingness to receive the set of SMS, and preferences regarding the total number of SMS to be sent, frequency, specific days of the week and hours of the day to receive them; (d) perceptions and motivations towards seeking mental health care, which contained the main topics used to develop the SMS contents.

The theoretical framework used for the questions regarding perceptions and motivations towards seeking mental health care was based on the Theory of Planned Behavior [28], and it explored three pairs of opposite topics: perceived barriers and facilitators to seek mental health care (evaluation of the behavior), opinions of people close to them who would and would not support the patient's seeking mental health care (subjective norm), and perceived positive and negative consequences of seeking specialized care (beliefs about the behavior).

A research assistant approached the patients in the waiting rooms of the health centers and asked them if they would be willing to answer the questionnaire, then if they accepted, proceeded to provide informed consent. They were handed an informed consent form where all the information related to their participation was detailed. The research assistant reviewed the document with the participant and clarified any doubts. If they agreed, the participant ticked a box in the informed consent form and kept a copy. Then, the research assistant began the interview.

#### Analysis

Participants’ answers to each question were transcribed to a matrix in Microsoft Excel. Then, similar answers were categorized and ranked in order of frequency. Based on this information, four reminder and 10 motivational SMS were created and reviewed by the research team. The reminder SMS aimed to provide information to the recipients about the psychology service's office hours in order for them to be able to get an appointment following the recommendation from the primary health care provider after a mental health screening. The motivational SMS aimed to encourage the recipients to follow the recommendation made by the health care provider to seek specialized mental health care. Of the 14 SMS created, a total of seven were selected by the research team to further undergo a content validation process.

### Phase 2: content validation of the SMS

#### Setting and participants

For the validation phase, we aimed to interview 24 patients, six per health service, attending public primary health care services similar to where the intervention of the project would be implemented. These patients had to be 18 years or older and be able to provide informed consent. In addition, the research team decided to balance the patients by gender, interviewing three men and three women in tuberculosis, HIV/AIDS and chronic diseases services, with the aim of reducing possible gender biases in the opinions of the SMS developed.

Inclusion criteria were the same as in phase 1 (see Phase 1: Design of the SMS, Setting and participants).

#### Guiding principles of the content validation process

Each of the seven individual SMS developed in the previous phase was subjected to a content validation process. The validation prioritized two aspects: understanding of the message, and appeal to potential recipients. Suggestions from the participants were collected in order to improve the individual SMS, considering also that the validated messages would become part of a cohesive set of SMS. The final set of SMS was reviewed and approved by the research team taking into account the input from potential recipients collected during this phase.

#### Data collection tools and procedures

A 6-item questionnaire was adapted from a previous SMS design and validation study developed by our group (see Supplementary Material 2) [29]. The questionnaire contained open- and close-ended questions. The first two questions asked about the understanding of the SMS contents, the following three questions asked about the SMS appeal, and the last question aimed to collect information from the patients on how the wording and contents of the text message could be improved. The seven SMS subjected to content validation were printed and handed over to the participants one by one. For each SMS, the participants had to answer the 6-item questionnaire.

The understanding of the set of SMS was assessed by asking the participant how they would explain the contents of the SMS to another person. In addition, participants were asked if any specific word or the way it was worded was not understood. Their answers and suggestions were annotated to improve the wording of the text messages.

To assess the appeal of the messages, participants were asked to rate each SMS with a score from 1 to 10, where 1 means they disliked the SMS the most and 10 means they liked the SMS the most. Moreover, participants were asked what they liked about the SMS and what they did not like, in order to evaluate if further changes needed to be made in terms of contents or messages conveyed.

Finally, an open-ended question asked about suggestions to improve the SMS, either an individual one or the whole set. These suggestions could be as general as making the SMS shorter or longer, or as specific as changing a word.

In addition, similar to the previous phase, a research assistant approached the patients in the waiting rooms of the health centers and asked them if they would be willing to answer the questionnaire, then if they accepted, proceeded to provide informed consent. Similarly to the design phase, they were handed an informed consent form which was reviewed by the research assistant to clarify any doubts. If they agreed, the participant ticked a box in the informed consent form and kept a copy. Then, the research assistant began the interview.

#### Analysis

The analysis of each SMS focused on the two main topics assessed by the validation questionnaire: understanding and appeal of the SMS contents. First, the research assistant and the communicator reviewed each of the participants’ answers. Then, the SMS was ranked based on the average appeal score. Next, difficulties with the understanding of the core message of the SMS and with specific words were reviewed. These difficulties coupled with the suggestions provided by the participants were used to improve the wording of the set of SMS. The results were presented to the research team and reviewed together. Finally, after careful consideration, the team reached a consensus on the five messages that would comprise the final set of SMS.

## Ethics

A study protocol detailing both the design and content validation phases was reviewed and approved by the local Institutional Review Board (IRB) of the Universidad Peruana Cayetano Heredia. All participants were asked to provide oral informed consent to be interviewed and for the recording of the audio, in the design phase. All interview audios were coded to avoid identification of the participant. In the content validation phase, participants were also asked to provide oral informed consent to answer the 6-item questionnaire.

## Results

### Participants’ characteristics

For phase 1, questionnaires were applied in three primary health care facilities. These facilities were public health centers in Lima, Peru. In these facilities, 39 patients out of the 40 projected answered the questionnaire (10 pregnant women, 10 patients with tuberculosis, 10 patients with hypertension and 9 patients with diabetes). Participants’ age ranged between 18 and 76 years old.

For phase 2, questionnaires were applied in the same three primary health care facilities but with the addition of a fourth health center, adding up to four primary health care centers. A total of 24 patients who attended prenatal, tuberculosis, HIV/AIDS, and chronic diseases services, six in each service, were interviewed. As planned, in each of the health services, except in the prenatal care service, three men and three women were interviewed. These participants were not the same as those interviewed in the design phase. Participants’ age ranged between 18 and 77 years old, with an average of 38 years.

### Patients’ phone literacy and willingness to receive SMS

Overall, most of the 39 patients who answered the questionnaire during phase 1 had sufficient knowledge regarding the use of SMS, with 92.3% owning and using a cellphone and 88.9% knowing how to open and read text messages. When asked about their willingness to receive texts messages that would indicate where to seek mental health care and motivate them to do so, 91.7% of the patients expressed they would like to receive the SMS, with the remaining 8.3% stating they do not like to receive text messages from strangers, among other reasons.

### Patients’ perceptions and motivations towards seeking mental health care

During phase 1, we collected information regarding the perceptions and motivations towards seeking mental health care. These perceptions and motivations were to be used as the main topics for the development of the set of SMS.

Firstly, we collected information about the people who would and would not support the patients if they were to seek psychological help. The answers were widely varied, and included different family members, such as spouses, parents, siblings, and also friends. Therefore, a common person across patients who could provide encouragement and support was not identified. This observation led to the research team deciding to not mention specific people (relatives or friends) in the SMS, in order to make the SMS content applicable to as many potential recipients as possible.

Secondly, we collected information concerning the barriers and facilitators to seek specialized mental health care. Regarding the barriers, the most common answers were the lack of time due to work or other activities and concerns about the costs related to getting an appointment with a mental health specialist. In terms of facilitators that would motivate the patients to engage in seeking mental health care, they mentioned to feel motivated to seek psychological help because they would have someone to talk to, they would receive guidance and feel better.

Thirdly, we collected information regarding the perceived positive and negative consequences of seeking mental health care. When asked about positive consequences, the four most common answers were feeling better, talking to someone about their problems, receiving guidance, and learning about themselves. Conversely, regarding the perceived negative consequences of seeking mental health care, most of the patients could not come up with an answer, with only one person mentioning there is a chance they might not develop a good relationship with the psychologist.

### Development of SMS contents

To develop the reminder messages, the research team decided to be specific as to where the recipient could find mental health care. Since all of the health centers where the *Allillanchu* Project was to be implemented had a psychology service, the SMS incorporated the name of the patient's health center and the working hours of this service.

For the motivational messages, the research team decided to use information from the barriers, facilitators, and positive consequences. Since interviewed patients found it hard to answer which possible negative consequences could result from seeking mental health care, this information was not used. The research team decided to develop two type of motivational messages: the first type would address the most common barriers mentioned by the patients, but also highlight positive consequences of seeking mental health care within the same SMS; the second type would solely focus on the positive consequences of seeking mental health care.

In addition, since the SMS were to be received after a positive screening for common mental disorders, the research team decided to avoid any reference to these disorders in order to avoid patients’ relatives or friends who could access their cellphones learning about the screening results.

Finally, in order to make the set of SMS as tailored as possible to the potential recipients, the text messages had a specific structure that would open with a tailored greeting using the name of the patient, then the specific contents of each SMS, a motivational phrase, and a closing mentioning the health center as the sender of the SMS. The final result was a set of seven SMS to be subjected to content validation during phase 2 (see Table 1).

**Table 1.** Structure of SMS validated

	Type of SMS [average appeal score]	Structure	Original content	English translation
1	Reminder [8.5 points]	Customized greeting + customized reminder of the health center's psychologist service hours + motivational phrase + Sender	Hola (nombre)! El psicologo de (centro de salud) te espera de lunes a viernes de 8 am a 2 pm. Buscalo! Tu centro de salud	Hi (name)! The psychologist of the (name of the health center) awaits for you from Monday to Friday from 8 am to 2 pm. Look for him! Your health center
2	Motivational [9.0 points]	Customized greeting + perceived barrier (lack of time) + importance of seeking help + motivational phrase + Sender	Hola (nombre)! Sabemos que a veces falta tiempo, pero tu bienestar es primero. Busca al psicólogo. Tu centro de salud	Hi (name)! We know sometimes we do not have time, but your wellbeing is first. Look for the psychologist. Your health center
3	Motivational [8.8 points]	Customized greeting + perceived barrier (costs of seeking help) + Motivational phrase + facilitator (free with health insurance) + Sender	Hola (nombre)! Sentirte bien no tiene precio. Busca al psicólogo, es gratis con tu seguro. Tu centro de salud	Hi (name)! Feeling good is priceless. Look for the psychologist, it is free with your health insurance. Your health center
4	Motivational [8.9 points]	Customized greeting + positive consequence of seeking help (feeling better after talking to the psychologist) + motivational phrase + Sender	Hola (nombre)! Conversar con el psicólogo te hará sentir tan bien como antes. Buscalo! Tu centro de salud	Hi (name)! Talking with the psychologist will make you feel as good as before. Look for him! Your health center
5	Motivational [9.5 points]	Customized greeting + motivation to seek help (having someone to talk to) + positive consequence (feeling better) + motivational phrase + Sender	Hola (nombre)! Todos necesitamos conversar de nuestras cosas. El psicólogo te escuchara y te sentiras mejor. Buscalo. Tu centro de salud	Hi (name)! We all need to talk about our issues. The psychologist will listen to you and you will feel better. Look for him. Your health center
6	Motivational [8.9 points]	Customized greeting + positive consequence of seeking help (feeling better after talking to the psychologist) + motivational phrase + Sender	Hola (nombre)! Los consejos del psicólogo te ayudaran a sentirte mejor. Buscalo. Tu centro de salud	Hi (name)! The psychologist's advices will help you feel better. Look for him. Your health center
7	Motivational [9.6 points]	Customized greeting + positive consequence of seeking help (receive guidance) + positive consequence (feeling better) + motivational phrase + Sender	Hola (nombre)! Con el psicólogo encontraras la orientación que necesitas. Visitalo, te hara sentir bien. Tu centro de salud	Hi (name)! With the psychologist you will find the guidance that you need. Visit him, it will make you feel better. Your health center

### Appeal of the set of SMS

During phase 2, as part of the content validation process, the interviewed patients scored each of the seven SMS developed. The appeal scores for the seven SMS ranged from 8.5 to 9.6 points out of 10 points, with an average score of 9.0 (SD±0.4) points.

The lowest rated SMS was the reminder SMS (SMS #1, Table 1), and the main reasons stated by the patients were that, most of the times, the psychologist did not comply with working hours mentioned in the SMS or that they were not able to attend during these hours. This is a limitation that goes beyond the objective of the SMS, which was to remind the patients they were recommended to seek psychological help and they are able to do so in the health center during specific working hours.

The second lowest rated SMS (SMS #3, Table 1) addressed the barrier of costs and mentioned that the consultation with the psychologist is free with their health insurance. Some people stated that it was not true, indicating limited awareness about the coverage of the government or social security's health insurances regarding mental health activities. Therefore, despite the low rating, it was decided to keep this content in order to inform patients their health insurance covers consultations with the psychologist.

The highest rated SMS (SMS #7, SMS #5, and SMS #2) had less negative opinions and objections to the content, they were well received by the interviewed patients who, when asked what they liked the most about these messages, highlighted the SMS mentioning the importance of putting your wellbeing first, talking about their problems and having someone who listens and can provide some guidance.

Based on the scores, the research team chose the five text messages that comprise the final set of SMS to be used during the study intervention. Since there were no significant suggestions to change the wording of the messages, the contents remained the same. The final set of text messages are shown in Table 2.

**Table 2.** Final set of SMS

	Original content	English translation
1	Hola (nombre)! El psicologo de (centro de salud) te espera de lunes a viernes de 8 am a 2 pm. Buscalo! Tu centro de salud	Hi (name)! The psychologist of the (name of the health center) awaits for you from Monday to Friday from 8 am to 2 pm. Look for him! Your health center
2	Hola (nombre)! Sabemos que a veces falta tiempo, pero tu bienestar es primero. Busca al psicólogo. Tu centro de salud	Hi (name)! We know sometimes we do not have time, but your wellbeing is first. Look for the psychologist. Your health center
3	Hola (nombre)! Sentirte bien no tiene precio. Busca al psicólogo, es gratis con tu seguro. Tu centro de salud	Hi (name)! Feeling good is priceless. Look for the psychologist, it is free with your health insurance. Your health center
4	Hola (nombre)! Todos necesitamos conversar de nuestras cosas. El psicólogo te escuchara y te sentiras mejor. Buscalo. Tu centro de salud	Hi (name)! We all need to talk about our issues. The psychologist will listen to you and you will feel better. Look for him. Your health center
5	Hola (nombre)! Con el psicólogo encontraras la orientación que necesitas. Visitalo, te hara sentir bien. Tu centro de salud	Hi (name)! With the psychologist you will find the guidance that you need. Visit him, it will make you feel better. Your health center

## Discussion

We applied a standardized methodology for the construction and content validation of a set of SMS, a key component of a mental health intervention implemented in public health services in Lima, Peru. The methodology used was centered on the potential recipients, where they were the key informants who provided essential information for the construction of the SMS contents and provided their opinions to validate the SMS. The use of the patients’ own experiences coupled with the use of their names and mentions of their health center allowed the SMS to be tailored to the potential recipients. Evidence suggests tailoring not only increase understanding of the contents provided in the set of SMS but are also more persuasive in promoting behavior change [30–32].

The set of SMS was designed to remind and motivate patients to follow a recommendation made by a primary health care provider, to seek specialized mental health care when a potential common mental disorder was detected. The SMS contents aimed to address some context-specific barriers patients usually face when following a recommendation by health care providers, such as lack of time to go to a consultation. In addition, the contents also aimed to provide information that may not be of common knowledge for the patients, specifically, consultations with the psychologists being free with health insurance. Addressing this barrier is important since according to the information collected from the patients, not having money to pay for a consultation is an important concern and a reason for not seeking help.

In the same vein, the SMS contents also highlighted positive outcomes that may stem from complying with the desired behavior, e.g. feeling good about themselves, having someone to talk to, and receiving guidance. Since these positive outcomes were collected from the potential recipients of the SMS, we increased the tailoring of the messages and allow the patients to weigh in the barriers versus the potential rewards of following the health providers’ recommendation.

Regarding the content validation process, patients were asked to score the SMS, four messages received between eight and nine points, and three messages received between nine and ten points (the highest scores). This high scoring signals that the messages were well received and easily understood by the patients.

Studies have typically used SMS to provide support messages, reminders, and information to patients [20, 33, 34], and as a result, most of the studies and reviews point out the importance of tailoring the contents of the SMS. Furthermore, most studies usually focus on the effectiveness of the intervention itself and do not give much information regarding the design and validation process of the SMS contents, which can provide insights to researchers in methods to tailor SMS contents to their target populations.

The few studies that give an account on how the SMS were developed show a tendency to focus either on the design or on the content validation of the SMS. The design is usually varied in methodology, some studies report the contents were developed by the research team, based on literature review [35, 36], and others are based on data collected from interviews or focus groups with the potential recipients of the SMS [37]. For the validation process, the methodology is usually qualitative, using focus groups [35, 37, 38] or interviews with potential recipients [35, 36].

The strength of this approach relies on the development of the SMS contents that was directly based on perceived barriers, facilitators, and positive consequences of seeking psychological help with direct input from the potential recipients attending the health services where the project's intervention would be implemented. In addition, an element of tailoring was featured in the SMS, including the participant's name in each SMS together with the health center they could visit. Finally, it is important to further explore the effectiveness of tailoring contents, and in the process, include a detailed account of the methodology used to develop them.

### Limitations

Amongst the limitations, the sample size was small in both the design and content validation phases. However, given the qualitative nature of the study, the focus was on the quality of the data collected rather than the quantity, and we manage to collect information from a diverse sample and made sure to involve patients from all the health services that were targeted in the *Allillanchu* project.

Furthermore, we cannot rule out some social desirability bias, particularly the willingness of the interviewees to express a positive opinion about the SMS, thus yielding very high scores for each SMS they were rating. Yet, the same reaction towards high rating was also observed in a previous multi-country study of validation of SMS [29], and the key appears to be to have a sufficient number of potential SMS to start with and move only a few of them to the validation stage, where appealing is explored.

## Conclusions

The final set of SMS developed and validated have followed a thorough approach that is likely to be applicable to other similar low-resource settings and the methodology used can be well adapted to develop SMS for other chronic conditions.

## Supplementary material

For supplementary material accompanying this paper visit http://dx.doi.org/10.1017/gheg.2017.18. click here to view supplementary material
