# Supplementary material for: Design and content validation of a set of SMS to promote seeking of specialized mental health care within the Allillanchu Project
Source: Glob Health Epidemiol Genom. 2018 Jan 31;3:e2. doi: 10.1017/gheg.2017.18 (PMC5870406; doi:10.1017/gheg.2017.18)
Supplement: Supplementary file 1 [file S2054420017000185sup001.zip › Supplementary material 2 - GHEG 20.09.2017.docx]

***Supplementary material 2. Validation phase questionnaire***

| **I. Interview data** | | | | | | |
| --- | --- | --- | --- | --- | --- | --- |
| **Participant ID** | |  | |  | | |
| **Health condition** | |  | | | | |
| **Health center** | |  | | | | |
| **Date** | |  | | | | |
| **Interviewer** | |  | | | | |
| **Place of interview** | |  | | | | |
| **Recording authorization** | | Yes No | | | | |
| **Interviewer notes** | | | | | | |
|  | | | | | | |
| **II. Demographic and socioeconomic data** | | | | | | |
| 1 | Gender | |  | | | |
| 2 | Age | |  | | | |
| 3 | Place of birth | |  | | | |
| 4 | Years of education | |  | | | |
| 5a | Duration of pregnancy (for pregnant women only) | |  | | | |
| 5b | Time of diagnosis (for patients with tuberculosis, HIV/AIDS, diabetes or hypertension) | |  | | | |
| 6 | Main ocupation in the last 6 months | |  | | | |
| **III. Phone literacy** | | | | | | |
| 7 | Do you own a cellphone? | |  | | | |
| 8 | Do you share the cellphone with another person? | |  | | | |
| 9ª | With whom do you share your cellphone? | |  | | | |
| 9b | What use this person gives to your cellphone? | |  | | | |
| 10 | Do you use your cellphone to… | | | | | |
|  | **Use** | | **Yes** | | **No** | **Doesn’t answer** |
| 10a | … sent text messages? | | 1 | | 2 | 99 |
| 10b | … receive text messages? | | 1 | | 2 | 99 |
| 10c | … make calls? | | 1 | | 2 | 99 |
| 10d | … receive calls? | | 1 | | 2 | 99 |
| 10e | … browse the internet? | | 1 | | 2 | 99 |
| **IV. SMS validation** | | | | | | |
| Show the participant the first SMS and ask the following questions. Repeat the procedure for each SMS | | | | | | |
| 1. Imagine you have to explain the content of the message to another person. What would you say? | | | | | | |
| 1. Which words do you not understand in the text message? | | | | | | |
| 1. Imagine that tomorrow you receive this text message in your cellphone. In a scale from 1 to 10, where 1 means you do not like the message at all and 10 means you like it a lot. Which score would you give this text message? | | | | | | |
| 1. What do you like from this text message? | | | | | | |
| 1. What do you NOT like from text message? | | | | | | |
| 1. Do you have any suggestion to improve this text message? (for instance, make it shorter or longer, change a word, replace a word, etc.) | | | | | | |
