# Supplementary material for: Design and content validation of a set of SMS to promote seeking of specialized mental health care within the Allillanchu Project
Source: Glob Health Epidemiol Genom. 2018 Jan 31;3:e2. doi: 10.1017/gheg.2017.18 (PMC5870406; doi:10.1017/gheg.2017.18)
Supplement: Supplementary file 1 [file S2054420017000185sup001.zip › Supplementary material 1 - GHEG 20.09.2017.docx]

***Supplementary material 1. Design phase questionnaire***

| **I. Interview data** | | | | | | |
| --- | --- | --- | --- | --- | --- | --- |
| **Participant ID** | |  | |  | | |
| **Health condition** | |  | | | | |
| **Health center** | |  | | | | |
| **Date** | |  | | | | |
| **Interviewer** | |  | | | | |
| **Place of interview** | |  | | | | |
| **Recording authorization** | | Yes No | | | | |
| **Interviewer notes** | | | | | | |
|  | | | | | | |
| **II. Demographic and socioeconomic data** | | | | | | |
| 1 | Gender | |  | | | |
| 2 | Age | |  | | | |
| 3 | Place of birth | |  | | | |
| 4 | Years of education | |  | | | |
| 5a | Duration of pregnancy (for pregnant women only) | |  | | | |
| 5b | Time of diagnosis (for patients with tuberculosis, HIV/AIDS, diabetes or hypertension) | |  | | | |
| 6 | Main ocupation in the last 6 months | |  | | | |
| **III. Phone literacy** | | | | | | |
| 7 | Do you own a cellphone? | |  | | | |
| 8 | Do you share the cellphone with another person? | |  | | | |
| 9ª | With whom do you share your cellphone? | |  | | | |
| 9b | What use this person gives to your cellphone? | |  | | | |
| 10 | Do you use your cellphone to… | | | | | |
|  | **Use** | | **Yes** | | **No** | **Doesn’t answer** |
| 10a | … sent text messages? | | 1 | | 2 | 99 |
| 10b | … receive text messages? | | 1 | | 2 | 99 |
| 10c | … make calls? | | 1 | | 2 | 99 |
| 10d | … receive calls? | | 1 | | 2 | 99 |
| 10e | … browse the internet? | | 1 | | 2 | 99 |
| 11 | On a regular week, from Monday to Sunday, how many text messages do you receive? | | None  Less than 10 messages  From 10 to 20 messages  More than 20 messages  Doesn’t know/doesn’t remember | | | |
| 12 | How easy it is for you to open and read a text message on your cellphone? | | Very easy, opens and reads them without problems  Has some difficulties to open or read them  Very hard, has difficulties to open and read them  Doesn’t know/does not receive text messages | | | |
| 13 | What problems do you have to open and/or read a text message? | | | | | |
| 14 | Sometimes we receive text messages from unknown numbers. What do you do with those messages? (Do you open and read them? Do you save them?) | | | | | |
| 15 | At any moment of the day, do you turn off your phone? | | No, I never turn it off  On the morning  On the afternoon  On the evening  At night | | | |
| **IV. Opinion on the project** | | | | | | |
| **Read to the participant:** “We are conducting a research project for people who have symptoms of depression, meaning they feel sad or down, to motivate them to go to the psychology service.  To accomplish this, we will send them text messages to their cellphone. These messages will remind them where they can have a consultation and the office hours of the service. The messages will also have phrases to motivate them to seek this consultation.  I want you to imagine you are feeling sad or down, and in your consultation with the health provider, he/she recommends you to go to the psychology service” | | | | | | |
| 16 | Would you like to receive text messages on your cellphone that informs you and motivate you to seek a consultation with the psychologist? | | Yes  No  Doesn’t answer | | | |
| 17 | We consider sending three text messages per week. Do you think it is very few, okay or too many? Why? | | | | | |
| 18 | We consider sending the text messages over a period of two weeks (three per week). Do you think two weeks is too short, okay or too much? Why? | | | | | |
| 19 | Which days of the week would you like to receive these text messages? | | Any day of the week  Only from Monday to Friday  Only weekends  Doesn’t know/doesn’t answer | | | |
| 20 | At what time of the day would you like to receive the text messages? | | Morning  Afternoon  Evening  Night  Doesn’t know/doesn’t answer | | | |
| **V. Motivations and perceptions** | | | | | | |
| **Read to the participant:** “I would like you to imagine you are one the people who is feeling sad or down, and your health provider has recommended you to go to the psychology service.  Now I would like to ask you some questions to know your opinion regarding seeking a consultation with the psychologist. There are no good or bad answers, we only want to know your opinion. Please be as honest as possible when answering.” | | | | | | |
| 21 | What could be the barriers or limitations that may difficult you seeking a consultation with the psychologist? | | | | | |
| 22 | What would facilitate, make it easier or motivate you to seek a consultation with the psychologist? | | | | | |
| 23 | Which persons in your life would support you to seek a consultation with the psychologist? | | | | | |
| 24 | Which persons in your life would NOT support you to seek a consultation with the psychologist? | | | | | |
| 25 | What positive consequences do you consider would come out of having a consultation with the psychologist? | | | | | |
| 26 | What negative consequences do you consider would come out of having a consultation with the psychologist? | | | | | |
